# A novel molten-salt electrochemical cell for investigating the reduction of uranium dioxide to uranium metal by lithium using *in situ* synchrotron radiation

**DOI:** 10.1107/S1600577517000625

**Published:** 2017-02-16

**Authors:** Leon D. Brown, Rema Abdulaziz, Rhodri Jervis, Vidal Bharath, Thomas J. Mason, Robert C. Atwood, Christina Reinhard, Leigh D. Connor, Douglas Inman, Daniel J. L. Brett, Paul R. Shearing

**Affiliations:** a Electrochemical Innovation Lab, Department of Chemical Engineering, UCL, London WC1E 7JE, UK; b Diamond Light Source, Harwell Science and Innovation Campus, Didcot, Oxfordshire OX11 0DE, UK

**Keywords:** cell design, molten-salt reduction, spent fuel reprocessing, energy-dispersive X-ray diffraction

## Abstract

A novel electrochemical cell has been designed and built to allow for *in situ* energy-dispersive X-ray diffraction measurements to be made during reduction of UO_2_ to U metal in LiCl–KCl at 500°C. The electrochemical cell contains a recessed well at the bottom of the cell into which the working electrode sits, reducing the beam path for the X-rays through the molten-salt and maximizing the signal-to-noise ratio from the sample. Lithium metal was electrodeposited onto the UO_2_ working electrode by exposing the working electrode to more negative potentials than the Li deposition potential of the LiCl–KCl eutectic electrolyte. The Li metal acts as a reducing agent for the chemical reduction of UO_2_ to U, which appears to proceed to completion. All phases were fitted using Le Bail refinement. The cell is expected to be widely applicable to many studies involving molten-salt systems.

## Abstract 2

Energy-dispersive X-ray diffraction was used to follow the reduction of UO_2_ to U in LiCl–KCl eutectic. A novel electrochemical cell was designed and constructed in order to follow molten-salt electrochemical investigations *in situ*.

## 1. Introduction   

The electrochemical reduction of metal oxides to their metal phase using molten-salt media has gained significant interest since the FFC Cambridge process was realised (Chen *et al.*, 2000 ▸). Many studies have been performed in order to understand the electro-reduction pathway (Alexander *et al.*, 2006 ▸; Schwandt & Fray, 2005 ▸). However, due to the high temperature at which molten-salt electrolytes typically operate, the *ex situ* analysis of electrodes may be problematic and affected, for example, by structural changes associated with large changes in temperature and the removal of water-soluble species during cleaning of electrodes (Bhagat *et al.*, 2010 ▸).

Metal-chloride molten salts may be deployed as electrolytes due to their high ionic conductivities and, furthermore, the high temperatures at which these systems operate (>523 K) also afford rapid electrode kinetics. During electro-reduction in molten-salt environments, the metal oxide to be reduced is made into the working electrode and a graphite (or metallic) rod is made into the counter electrode. Upon polarizing the working electrode to sufficiently negative potentials, the reduction of these metal oxides may occur, liberating O^2−^ ions which are then transported through the electrolyte to the anode where it is oxidized to CO_2_, CO or O_2_ (depending on the material of the anode). In typical laboratory setups, this configuration is usually contained within a glassy carbon, ceramic or glass crucible.

Spent nuclear fuel reprocessing using molten salts was first demonstrated in the 1970s using the pyro-processing technique for the processing of integral fast reactor fuels using cadmium chloride to reduce metallic spent fuel in lithium chloride–potassium chloride eutectic (LKE), followed by electro-refining (Pierce *et al.*, 1993 ▸; Laidler, 1993 ▸; Laidler *et al.*, 1997 ▸). A similar pyrochemical technique has been investigated in Li_2_O-doped LiCl, which chemically reduces spent fuel oxides by electrochemically plating Li onto the spent fuel electrode (Hur *et al.*, 2003 ▸; Seo *et al.*, 2006 ▸; Jeong *et al.*, 2010 ▸; Kurata *et al.*, 2004 ▸). More recently, a *pyro-electrochemical* technique has been investigated which aims to electrochemically reduce spent fuel oxides, followed by selective electrodeposition in LKE (Brown *et al.*, 2013 ▸, 2015 ▸; Hu *et al.*, 2014 ▸). The focus of this paper is to demonstrate the experimental design and methodology developed to study the metal/molten-salt system using energy-dispersive X-ray diffraction. Here, our studies focus on the chemical reduction of UO_2_ by plating Li onto the working electrode in molten LKE at 450°C which is characterized *in situ* using synchrotron radiation.

*In situ* neutron diffraction and X-ray diffraction are valuable tools for identifying crystalline phases in the actual environment in which processes occur. For example, many *ex situ* studies observed the formation of Ti_3_O_5_ as an intermediate phase during the electrochemical reduction of TiO_2_ to Ti in the FFC Cambridge process (Dring *et al.*, 2005 ▸; Schwandt & Fray, 2005 ▸; Alexander *et al.*, 2006 ▸). However, during *in situ* X-ray diffraction, this phase was not observed (Bhagat *et al.*, 2010 ▸). Furthermore, in a recent *in situ* study the electrochemical reduction of UO_2_ to U in molten LKE was shown to occur in a single, four-electron transfer, process and occurred at a potential marginally more positive than the potential for Li deposition from the electrolyte (Brown *et al.*, 2013 ▸, 2015 ▸).

In this work, the design processes and considerations for the novel electrochemical cell used by Brown *et al.* (2015 ▸) are described in order to combine electrochemical measurements with *in situ* energy-dispersive X-ray diffraction. The authors also present a second electrochemical reduction mechanism: the chemical reduction of UO_2_ to U from electroplated Li metal from the electrolyte. The cell design presented is expected to fulfil a broader application for *operando* experimentation of molten-salt systems.

## 2. Materials and methods   

**Figure 1:**
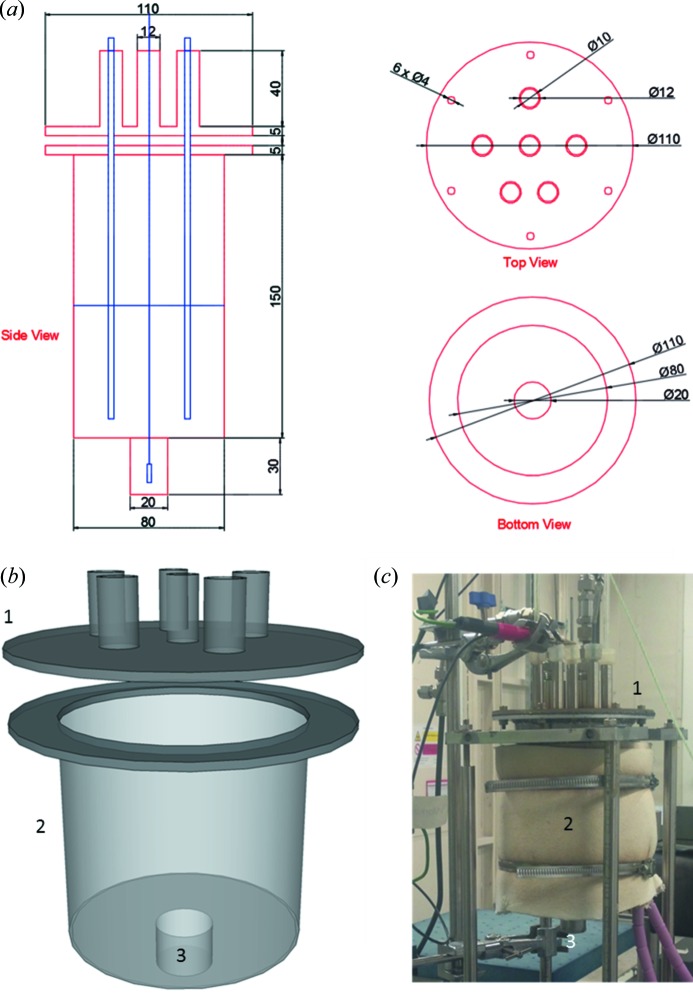
(*a*) CAD drawing of the electrochemical cell and electrochemical cell head used for experimentation. All dimensions stated are in mm. (*b*) Three-dimensional schematic of the electrochemical cell and (*c*) photograph of the electrochemical cell setup on the beamline. 1: electrochemical cell head. 2: electrochemical cell. 3: ‘well’ in which the working electrode is positioned. Under operation, LKE fills approximately half of the vessel, as shown in blue in (*a*). The positions of ancillary electrodes and thermocouple are also shown in blue.

A novel electrochemical cell, containing a ‘well’ at the bottom (where the working electrode is located), was manufactured from aluminium due to its low attenuation of X-rays. Incorporation of the well limited the X-ray beam path length through the sample environment, thus reducing attenuation by the structural components of the cell, and the molten-salt media. This would increase the signal-to-noise ratio of the diffraction pattern and simplify the sample alignment. The balance-of-apparatus components (counter electrode, reference electrode and thermocouple) are contained within the main body of the vessel, as shown in Fig. 1 ▸. All wall thicknesses of the aluminium cell are 3 mm.

The cell head was manufactured from 316 stainless steel, chosen due to its lower thermal conduction coefficient compared with aluminium. This then aids in desired cooling of the cell head. (In addition, stainless steel is easy to weld and reduces production time and cost, compared with aluminium.) The cell head also incorporated six hollow, open-ended, cylinders which were welded on top of the head. These were included to help ensure that the three electrodes, thermocouple and gas lines were held vertically (which helped to avoid possible short-circuiting of electrodes with the cell wall) and also acted as heat-transfer fins, again aiding cooling of the electrodes. The electrodes were held into the cell head cylinders *via* the use of high-temperature silicone rubber bungs. Electrical connections were made with a potentiostat by the use of crocodile clips crimped to the electrodes directly (see Fig. 1*c* ▸). The cell head also incorporated Swagelok fittings with non-return valves to allow for gas flow through the cell and to ensure no atmospheric gas would enter the cell during transportation from assembly in an argon-filled glove box.

A graphite gasket was also used to ensure a gas-tight fit between the stainless steel cell head and aluminium flange. The cell head was attached to the cell by the use of six M4 nuts and bolts located at angles of 60° from the centre of the vessel. A computer-aided-design (CAD) drawing of the electrochemical cell may be seen in Fig. 1 ▸.

The electrochemical cell was assembled inside an argon-filled glove box which maintained an oxygen and moisture composition of <0.1 p.p.m. 300 g of dried LKE (>99.9%, Sigma-Aldrich) was loaded into the electrochemical cell, along with the electrodes and a glass-sheathed K-type thermocouple. The thermocouple was located 2 mm above the bottom of the electrochemical cell. Note that, due to local rules within the synchrotron buildings, the UO_2_ working electrode was unable to be loaded into the electrochemical cell inside the glove box and was required to be inserted into the cell on the beamline itself. The Suba-Seal design allowed for this to be achieved with a gas-tight seal.

A heating jacket was then secured to the electrochemical cell and connected to the PID-controlled power source. The heating jacket was a custom-built flexible ceramic pad heater (Artech Services, UK) powered by a three-phase transformer. The PID controller input was a single glass-sheathed K-type thermocouple which was immersed in the molten salt, which provided a constant, stable temperature reading at operating temperature. A layer of ceramic-based, flexible, furnace-grade insulation was also wrapped around the heating jacket. The insulation and heating jacket were fixed to the cell *via* a number of jubilee clips.

The cell was heated at a ramp rate of 10°C min^−1^ from room temperature to a working temperature of 500°C, as recorded by the thermocouple. Once the working temperature was achieved, pre-electrolysis (see §3 for more details) was performed for 2 h to remove any electro-active contaminants from the molten salt. Once the electrochemical cell was secured onto the beamline sample stage, the gas line (research grade, <0.5 p.p.m. H_2_O and O_2_) was purged and connected to the electrochemical cell and the non-return valves were opened. The outlet gas was pumped through a series of gas traps to absorb any chlorine gas that may form during electrochemical measurements. The gas trap comprised a sequence of four Dreschel bottles: the first was empty to ensure no liquid flowed into the cell’s gas outlet stream due to any back pressure; the second and third bottles contained NaOH solutions to absorb chlorine-containing vapours from the cell, and the last bottle was de-ionized water.

During experimentation, a metallic cavity electrode (MCE) filled with UO_2_ powder was employed as the working electrode. MCEs are fabricated by drilling a 0.5 mm-diameter hole into a 0.5 mm-thick metallic foil and are described elsewhere (Qiu *et al.*, 2005 ▸; Rao *et al.*, 2007 ▸). This study used a 2.5 mm × 15 mm × 0.5 mm (W × L × D) Mo foil (>99.9%, Alfa Aesar), into which five 0.5 mm-diameter holes were drilled. Pure UO_2_ powder was prepared (Brown *et al.*, 2015 ▸) and pressed into the five MCE holes. The MCE was attached to a 3 mm-diameter 250 mm-long Mo rod using 0.2 mm-diameter Mo wire (rod and wire both >99%, Alfa Aesar). This configuration was made the working electrode. A three-electrode setup was employed using a dense graphite rod (3.18 mm-diameter, Alfa Aesar) counter electrode and an all glass Ag/AgCl reference electrode. All electrodes were held in place using silicone Suba-Seals (Sigma-Aldrich) which also allow for gas-tight sealing to be achieved.

The electrochemical cell here aims to achieve direct *in situ* characterization of electrochemical processes in molten-salt media, similar to that achieved by Styles *et al.* (2012 ▸) and Jackson *et al.* (2010 ▸). However, the furnace designs used in those studies used much more expensive materials, a result of the much higher temperatures required to heat CaCl_2_. In contrast, the LKE system operates at temperatures much lower than that of the melting point of aluminium which permits its use as a material of construction. In addition, the cell described in this work also permits the use of very small sample quantities in the form of MCE electrodes which allow for rapid completion of reactions and a reduction in any overpotential associated with metal oxide powder pellet electrode configurations.

Energy-dispersive X-ray diffraction (EDXD) measurements were performed on beamline I12 (JEEP) at Diamond Light Source in the UK (Drakopoulos *et al.*, 2015 ▸). This diffraction technique differs from conventional, angular-dispersive, X-ray diffraction as the *d*-spacing is derived by determination of the wavelength of the diffracted polychromatic photons [as opposed to being derived from the diffraction angle of monochromatic photons (Kämpfe *et al.*, 2005 ▸)]. The diffraction angle may be kept constant by the use of two collimating slits and, as a direct result, a lozenge-shaped gauge volume is defined in space whereby only photons that are scattered from within this volume will be detected by the EDXD detector. The gauge volume is calculated to be 0.3 mm × 0.3 mm × 7.075 mm, using the method described by Rowels *et al.* (2011 ▸). This technique is particularly suited to the design of the electrochemical cell as the volume of the molten salt inside the well may be designed to be equal to the volume of the lozenge-shaped gauge volume. Placing the sample within this gauge volume has the advantage of being able to eliminate the signal from balance-of-apparatus components (the electrochemical cell, for example) and ensures that a high signal-to-noise ratio from the working electrode is collected. This is a useful advantage when using small samples, such as MCEs, which typically contain sub-milligrams of active material.

**Figure 2:**
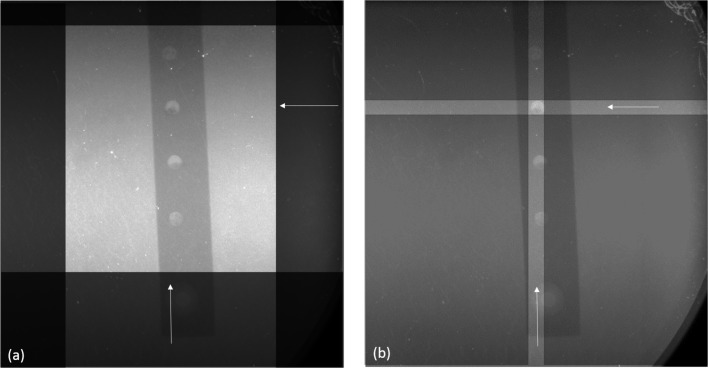
X-ray radiographs of the MCE in the well of the electrochemical cell showing *X–Y* alignment. (*a*) The MCE with the slits open and (*b*) complete alignment of the UO_2_-filled hole.

The beamline sample stage allowed translations in three dimensions with a 10 µm resolution. This ensured that the cell could be aligned in each direction by remotely moving the stage; the MCE was aligned in the *X* and *Y* directions using an X-ray imaging detector (which was removed during EDXD measurements). Fig. 2 ▸ shows X-ray images of a UO_2_-filled MCE in the electrochemical cell. *X*–*Y* alignment was achieved by moving the X-ray slits over a UO_2_-filled hole using live images. This alignment procedure had to be repeated for each new sample that was inserted into the cell. The slits created a 0.3 mm × 0.3 mm square X-ray beam which helped to increase the signal-to-noise ratio of UO_2_. For alignment in the *Z*-direction, it was necessary to move the MCE into the middle of the lozenge-shaped gauge volume. This was achieved by scanning the stage’s position along the X-ray beam direction (*Z*-direction) to optimize the signal-to-noise ratio of UO_2_ observed by the EDXD detector.

During experimentation, a 0.3 mm × 0.3 mm polychromatic X-ray beam of energies ranging from 45 to 150 keV irradiated a single UO_2_-filled cavity on the MCE working electrode, inside the electrochemical cell. EDXD data were collected with a cryogenically cooled 23-element, high-purity, germanium detector (Canberra Industries). The 23 detector elements are spaced every 8.18°, allowing azimuthal angles from 0° to 180° to be covered. The EDXD data collection was synchronized with the electrochemical measurements using an exposure time of 10 s. The take-off angle of 4.5° was defined by the collimating slits; all other X-rays which are diffracted at angles not equal to 4.5° were not detected. The collected diffraction patterns over the 23 elements were then averaged to produce a powder-averaged diffraction pattern, improving the data quality. The resulting EDXD data are plotted as a function of the photon energy of the diffracted X-rays, in contrast to angular-dispersive X-ray diffraction data that are obtained at a single wavelength and plotted as a function of the scattering angle, 2θ. All data were fitted using the Le Bail refinement. The Reitveld modelling approach was considered but was not used as it would have added extra complications that were not necessary for a relatively complex system studied here. Also, the likelihood of sample movement was relatively high (due to the volume change associated with reduction) which would have modified the absorption characteristics which need to be carefully considered (Rowles *et al.*, 2012 ▸; Scarlett *et al.*, 2009 ▸).

## 3. Experimental   

During experimentation, the electrochemical cell was heated to 500°C and left to thermally stabilize for 1 h. Pre-electrolysis was performed for 2 h to remove any electro-active species in the salt. For this, a molybdenum rod (>99%, Alfa Aesar) working electrode was held 200 mV more positive to the Li deposition potential, until the current fell to a value < 10 mA. After pre-electrolysis, the molybdenum rod was manually removed from the salt, after which a fresh UO_2_-filled MCE was inserted into the electrochemical cell. The cell was aligned to the centre of the incident X-ray beam in the *X* and *Y* axes using the translation sample stage and imaged using X-ray radiography. The cell was then aligned in the *Z*-axis by optimizing the peak intensities of UO_2_ at different positions along the incident X-ray beam using the EDXD detector. After alignment, continuous EDXD measurements were taken before, during and after exposing the UO_2_ MCE to potentials more negative than the Li deposition potential of the LKE electrolyte. Due to this potential, Li^+^ would be reduced to Li metal *via* equation (1) and plate onto the working electrode. The Li metal then chemically reduces UO_2_ *via* equation (2),
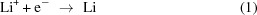


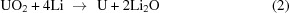
The peak intensities for U and UO_2_ were recorded every minute on each of the 23 EDXD detector elements and averaged to produce a single powder averaged diffraction pattern.

## 4. Results and discussion   

**Figure 3:**
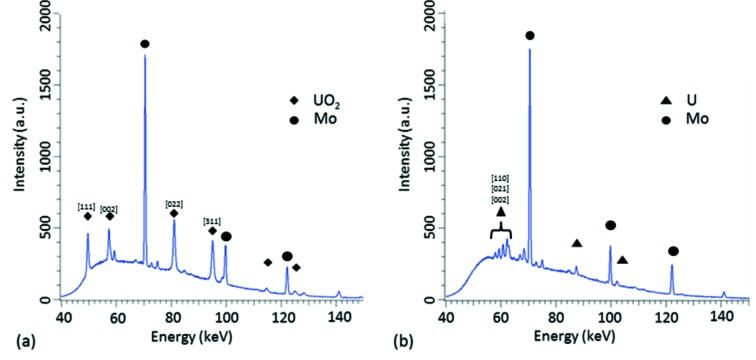
Sample single powder averaged diffraction patterns obtained (*a*) before Li deposition and (*b*) after Li deposition. Unmarked peaks are either minor peaks or Mo/Al fluorescence peaks. The *hkl* values for selected peaks are shown in square brackets.

Sample averaged powder diffraction patterns obtained from continuous EDXD measurements are presented in Fig. 3 ▸ before and after Li deposition onto the UO_2_ working electrode.

In Fig. 3(*a*) ▸, a prominent peak exists corresponding to the intensities of UO_2_ and Mo obtained by EDXD measurements on the UO_2_ working electrode. After Li deposition, the peak intensities of UO_2_ disappear and peaks characterizing U metal appear; Video S1 in the supporting information shows the transition from UO_2_ to U metal during the continuous EDXD measurements. From this, the point of Li deposition is easily observed as the peak intensities of UO_2_ all reduce whilst those for U metal appear. It is also possible to observe from Fig. 3 ▸ and Video S1 that the peak intensities for UO_2_ fall to un­observable levels, indicating full chemical reduction of UO_2_ to U. A total of 5.7 C was passed during Li plating, which would result in a mass of ∼0.4 mg of deposited Li. From equation (2), this amount of Li would allow for 0.1 mg of UO_2_ to be reduced. The MCE electrodes hold approximately 0.04 mg of UO_2_ powder (Brown *et al.*, 2015 ▸) and results in an efficiency of 40%. This efficiency is likely to be heavily affected by the fact that Li metal exists in the liquid state under the conditions employed.

**Figure 4:**
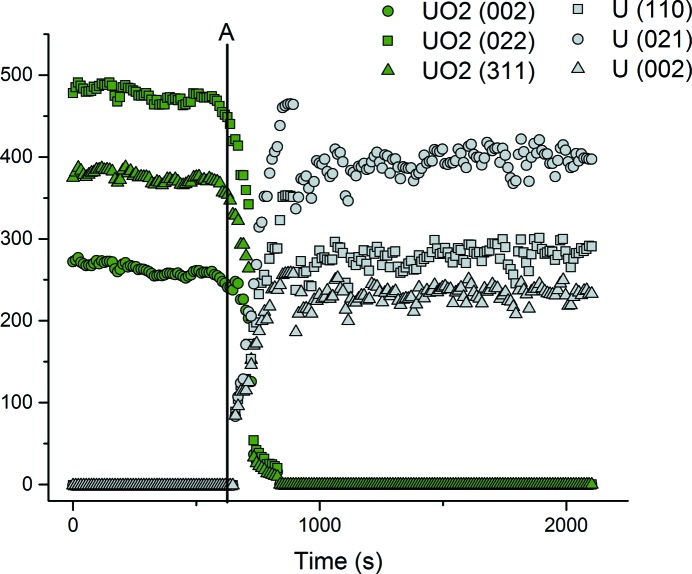
Peak intensities for different crystal planes of UO_2_ and U metal *versus* time during continuous EDXD measurements. The line denoted ‘A’ depicts the point that Li deposition occurred, at ∼650 s.

Moreover, Fig. 4 ▸ shows the change of peak intensity for the prominent peak crystal planes for both UO_2_ and U metal *versus* time. Peak intensities for the U phase start at zero, while peaks for UO_2_ are all observable. After ∼650 s, the cell is subjected to potentials more negative than the decomposition potential of the LKE electrolyte for 10 s. At this point, Li metal would be electrochemically plated onto the UO_2_-filled working electrode. After Li has been plated onto the electrode, the peak intensities for U begin to increase as the peak intensities for UO_2_ begin to decrease. In addition, the peak intensities for each observable plane for UO_2_ are all seen to fall to a value of zero. This is indicative of the chemical reaction proceeding to completion. The intensity of the diffraction peaks of U is seen to decrease slightly at ∼900 s and is most likely due to small amounts of powder falling out of the MCE due to the volume change associated with the reduction process (Brown *et al.*, 2016 ▸). In the context of pyroprocessing, this oxide reduction process could potentially be performed inside the electro-refiner unit, negating the use of the direct oxide reduction unit. The use of the electrochemical cell described in this work enabled high signal-to-noise ratios to be attained, as shown in Fig. 3 ▸.

## 5. Conclusions   

A novel electrochemical cell has been constructed in order to allow for *in situ* energy-dispersive X-ray diffraction to follow the reduction of UO_2_ to U in molten lithium chloride–potassium chloride eutectic. Peak intensities for UO_2_ are all seen to reduce to unobservable levels simultaneously as peak intensities for U metal increase, suggesting the reaction goes to completion, as expected. The electrochemical cell allowed for sub-milligram samples of powder to be studied in a high-temperature molten-salt environment due to its inherent design: the material of construction and the ‘well’ both substantially reduce X-ray attenuation, resulting in high signal-to-noise ratios. The use of sub-milligrams of powder allowed for low current responses and, thus, lower voltage drop due to ohmic losses (iR) to be studied, increasing the accuracy of electrochemical experimentation. The cell design is also expected to be applied to other molten-salt systems and to different geometries of electrodes, including pellet and thin-film electrodes.

## Supplementary Material

Video S1: Time-lapse diffraction datashowing the change in EDXD signal during Li electroplating. DOI: 10.1107/S1600577517000625/mo5149sup1.mov

## References

[bb1] Alexander, D. T. L., Schwandt, C. & Fray, D. J. (2006). *Acta Mater.* **54**, 2933–2944.

[bb2] Bhagat, R., Dye, D., Raghunathan, S. L., Talling, R. J., Inman, D., Jackson, B. K., Rao, K. K. & Dashwood, R. J. (2010). *Acta Mater.* **58**, 5057–5062.

[bb3] Brown, L. D., Abdulaziz, R., Jervis, R., Bharath, V. J., Atwood, R. C., Reinhard, C., Connor, L. D., Simons, S. J. R., Inman, D., Brett, D. J. L. & Shearing, P. R. (2015). *J. Nucl. Mater.* **464**, 256–262.

[bb4] Brown, L. D., Abdulaziz, R., Simons, S., Inman, D., Brett, D. J. L. & Shearing, P. R. (2013). *J. Appl. Electrochem.* **43**, 1235–1241.

[bb5] Brown, L. D., Abdulaziz, R., Tjaden, B., Inman, D., Brett, D. J. L. & Shearing, P. R. (2016). *J. Nucl. Mater.* **480**, 355–361.

[bb6] Chen, G. Z., Fray, D. J. & Farthing, T. W. (2000). *Nature (London)*, **407**, 361–364. 10.1038/35030069 11014188

[bb7] Drakopoulos, M., Connolley, T., Reinhard, C., Atwood, R., Magdysyuk, O., Vo, N., Hart, M., Connor, L., Humphreys, B., Howell, G., Davies, S., Hill, T., Wilkin, G., Pedersen, U., Foster, A., De Maio, N., Basham, M., Yuan, F. & Wanelik, K. (2015). *J. Synchrotron Rad.* **22**, 828–838. 10.1107/S1600577515003513 PMC4416690 25931103

[bb8] Dring, K., Dashwood, R. & Inman, D. (2005). *J. Electrochem. Soc.* **152**, E104–E113.

[bb9] Hu, D., Stevenson, A. & Chen, G. Z. (2014). *ECS Trans.* **64**, 585–592.

[bb10] Hur, J.-M., Seo, C.-S., Hong, S.-S., Kang, D.-S. & Park, S.-W. (2003). *React. Kinet. Catal. Lett.* **80**, 217–222.

[bb11] Jackson, B., Dye, D., Inman, D., Bhagat, R., Talling, R., Raghunathan, S., Jackson, M. & Dashwood, R. (2010). *J. Electrochem. Soc.* **157**, E57–E63.

[bb12] Jeong, S. M., Shin, H.-S., Hong, S.-S., Hur, J.-M., Do, J. B. & Lee, H. S. (2010). *Electrochim. Acta*, **55**, 1749–1755.

[bb13] Kämpfe, B., Luczak, F. & Michel, B. (2005). *Part. Part. Syst. Charact.* **22**, 391–396.

[bb14] Kurata, M., Inoue, T., Serp, J., Ougier, M. & Glatz, J.-P. (2004). *J. Nucl. Mater.* **328**, 97–102.

[bb15] Laidler, J. J. (1993). Presented at *GLOBAL’93 – International Conference on Future Nuclear Systems: Emerging Fuel Cycles and Waste Disposal Options*, 12–17 September 1993, Seattle, WA, USA.

[bb16] Laidler, J. J., Battles, J. E., Miller, W. E., Ackerman, J. P. & Carls, E. L. (1997). *Prog. Nucl. Energy*, **31**, 131–140.

[bb17] Pierce, R. D., Johnson, T. R., McPheeters, C. C. & Laidler, J. J. (1993). *JOM*, **45**, 40–44.

[bb18] Qiu, G., Ma, M., Wang, D., Jin, X., Hu, X. & Chen, G. Z. (2005). *J. Electrochem. Soc.* **152**, E328.

[bb19] Rao, K., Brett, D., Inman, D. & Dashwood, R. J. (2007). *Proceedings of the 11th World Conference on Titanium (Ti-2007)*, 3–7 June 2007, Kyoto, Japan. The Japanese Institute of Metals.

[bb20] Rowles, M. R. (2011). *J. Synchrotron Rad.* **18**, 938–941. 10.1107/S0909049511033267 21997921

[bb21] Rowles, M. R., Styles, M. J., Madsen, I. C., Scarlett, N. V. Y., McGregor, K., Riley, D. P., Snook, G. A., Urban, A. J., Connolley, T. & Reinhard, C. (2012). *J. Appl. Cryst.* **45**, 28–37.

[bb22] Scarlett, N. V. Y., Madsen, I. C., Evans, J. S. O., Coelho, A. A., McGregor, K., Rowles, M., Lanyon, M. R. & Urban, A. J. (2009). *J. Appl. Cryst.* **42**, 502–512.

[bb23] Schwandt, C. & Fray, D. J. (2005). *Electrochim. Acta*, **51**, 66–76.

[bb24] Seo, C. S., Park, S. B., Park, B. H., Jung, K. J., Park, S. W. & Kim, S. H. (2006). *J. Nucl. Sci. Technol.* **43**, 587–595.

[bb25] Styles, M. J., Rowles, M. R., Madsen, I. C., McGregor, K., Urban, A. J., Snook, G. A., Scarlett, N. V. Y. & Riley, D. P. (2012). *J. Synchrotron Rad.* **19**, 39–47. 10.1107/S0909049511039124 22186642

